# Enhancement of photo-driven biomethanation under visible light by nano-engineering of *Rhodopseudomonas palustris*

**DOI:** 10.1186/s40643-021-00383-5

**Published:** 2021-04-18

**Authors:** Meng-Yuan Chen, Zhen Fang, Li-Xia Xu, Dao Zhou, Xue-Jin Yang, Hu-Jie Zhu, Yang-Chun Yong

**Affiliations:** Biofuels Institute, School of Environment and Safety Engineering, Jiangsu University, 301 Xuefu Road, Zhenjiang, 212013 China

**Keywords:** Biomethanation, Carbon dioxide, Photocatalysis, *Rhodopseudomonas*, Quantum dot

## Abstract

Biomethanation is of great interest as it can transform CO_2_ to methane under ambient conditions. In particular, genetically engineered bacterium of *Rhodopseudomonas palustris* showed great promise for one-step biomethanation powered by solar energy, which is attractive for CO_2_ fixation as well as solar energy storage. However, biomethanation with *R. palustris* under visible light is inefficient due to its poor visible light response. In this study, CdS quantum dots with excellent visible light response were prepared and *R. palustris*/CdS hybrid cells were constructed. Interestingly, this bio-nano-hybrid cells showed high cell viability without significant cell damage, and the biomethanation performance of was enhanced about ~ 79% compared to that of the bare *R. palustris* cells. Moreover, the effects of different parameters on the methane production of this bio-nano-hybrid cells were determined, and the methane production rate was further improved by parameter optimization. This work demonstrated an efficient approach to reinforce the biomethanation of bacteria under unfavorable light wavelength, which would be helpful to extend the light spectra for photo-driven biomethanation.

**Supplementary Information:**

The online version contains supplementary material available at 10.1186/s40643-021-00383-5.

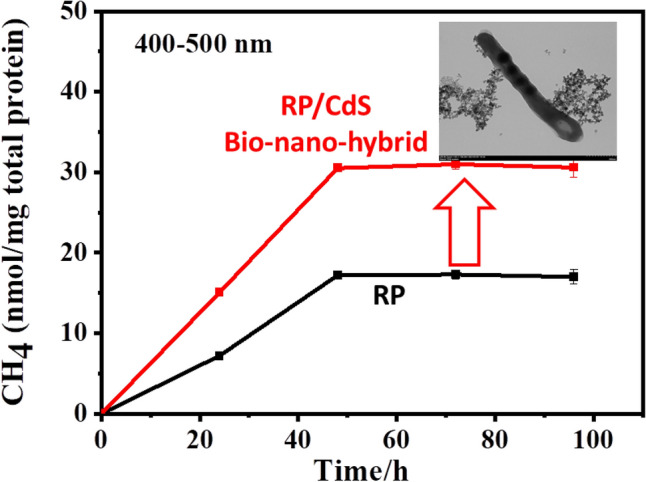

## Introduction

CO_2_ methanation which refers to transformation of CO_2_ to methane attracted much attention, as it can fix the greenhouse gas of CO_2_ and produce the renewable energy of methane (Zheng et al. 2018; Ma et al. 2020a, b). To date, several approaches have been developed for CO_2_ methanation, which include electrochemical reduction, photocatalysis, biomethanation, etc. (Nikiforov et al. 2020; Wang et al. 2017a, b; Ferry 2011). Among these methods, biomethanation which uses the microorganism as the catalytic module holds great promise because it required minimum energy and operation investment. Actually, there are at least one billion tonnes of methane produced with biomethanation each year (Thauer and Shima 2006). Methanogenic archaea, which had complex pathways for methanation, was the main microorganism for methane production from CO_2_ in nature (Zheng et al. 2018).

Recently, a new pathway for methane production by bacterial Fe-only nitrogenase was explored (Fixen et al. 2016; Zheng et al. 2018). It was found that the Fe-only nitrogenase from *Rhodopseudomonas palustris* could reduce CO_2_ to methane simultaneously with nitrogen fixation. More interestingly, 9% of diverse nitrogen-fixing microorganisms contained this Fe-only nitrogenase, suggesting this new biomethanation pathway is widespread among the microorganisms (Zheng et al. 2018; Fixen et al. 2018). This pioneering finding implied the possibility to use nitrogen-fixing bacteria for biomethanation. As compared with the methane production by methanogenic archaea, the biomethanation with bacteria (such as *R. palustris*) might have the advantages of fast growth rate, high catalytic activity, and easy operation. In particular, *R. palustris* can use solar energy to power this biomethane production, which is more sustainable and can be considered as another new route for solar energy storage. Therefore, photo-driven biomethanation with *R. palustris* attracted much attention (Fixen et al. 2016; Ma et al. 2020a, b). Recently, our group found that genetic engineering of a fast growth *R. palustris* strain (CGMCC 1.2180) resulted in efficient one-step photo-driven biomethanation from CO_2_ (Ma et al. 2020a, b). This photo-driven biomethanation was conducted under infrared light due to *R. palustris* has weak response under visible light. However, according to the wavelength and energy spectra of the solar light, the energy under visible light is much more powerful than that in the infrared region. Thus, it is desirable to improve the photo-driven biomethanation efficiency of *R. palustris* under visible light irradiation.

Bio-nano-hybrid cells, which integrated the advantages of living cell (high enzymatic catalytic activity and versatile/flexible metabolic pathways) with the exceptional properties of nanomaterials (excellent photocatalytic activity) (Yong et al. 2014; Saldrnoto et al. 2017; Yu et al. 2020; Luo et al. 2021), enabled the invention of photo-driven system for solar chemicals synthesis (Sakimoto et al. 2016; Guo et al. 2018; Shen et al. 2020; Wang et al. 2017a, b; Wei et al. 2018; Ye et al. 2020; Chen et al. 2019). For example, by integrating the Cu_2_O and *Shewanella oneidensis* MR-1, the bio-nano-hybrid cells showed high performance of solar hydrogen production (Shen et al. 2020). It was also explored that the photogenerated electrons from the nanomaterials could be injected into cells and were employed as the intracellular reducing equivalents for enzymatic catalysis (Shen et al. 2020; Zhang et al. 2020). Recently, the bio-nano-hybrid for biomethanation with methanogenic archaea was also developed, which improved the methane production by archaea (Ye et al. 2020). However, the methanogenic archaea employed a multi-step methanation pathway, which was much more complex than the one-step biomethanation of *R. palustris* (Ma et al. 2020a, b). Thus, it is speculated that the bio-nano-hybrid cells for biomethanation with bacteria of *R. palustris* could be developed.

With the aim of improving the performance of one-step photo-driven biomethanation, we developed bio-nano-hybrid cells based on the genetically engineered bacterial strain of *R. palustris* CGMCC 1.2180. The developed bio-nano-hybrid cell was characterized in details and the biomethanation performance was evaluated. It was found that the nano-engineering approach significantly improved the methane production of the strain under visible light and the effects of different parameters on methane production were determined.

## Materials and methods

### Bacterial strain and cultivation

The genetically engineered strain of *R. palustris* CGMCC 1.2180 (*nifD*^V75AH201Q^) (RP strain) was used in this study. The strain was anaerobically cultured in YP medium at 30 °C under shaking (200 rpm) (Ma et al. 2020a, b). When the OD_660_ reached 0.2, the cells were centrifuged (4000 rpm, 10 min) and transferred into anaerobic UPM medium (NFM medium (Zhang et al. 2018) with 1 g/L yeast extract and 3 g/L sodium acetate) for cell growth and nitrogenase accumulation under 60 W LED lamp at 30 °C.

### CdS quantum dots (QDs) synthesis

The CdS QDs were synthesized according to previous report with minor modification (Ding et al. 2019). Briefly, CdO (38.4 mg), oleic acid (OA, 1904 µl) and 1-octadecene (ODE, 10 g) were added into a 100-ml flask and heated to 300 °C under argon. Then, the sulfur precursor (0.048 g sulfur powder dispersed in 10 ml ODE) was injected into the flask and the mixture was cooled down to 250 °C and kept for 1 h to allow the growth of CdS QDs. After that, the unreacted reagents were extracted with methanol and the CdS QDs was precipitated by chloroform and acetone according to previous report (Ding et al. 2019).

### CdS QDs ligand exchange

The ligand exchange of CdS QDs was performed according to previous report (Ding et al.^2019^). A mixture of 1 ml 3-mercaptopropionic acid, 3 ml CdS QDs (dissolved in chloroform) and 3 ml ethanol was vigorously stirred and heated for 1 min (40 °C). Then, 10 ml NaOH (1 M) was added into the mixture and stirred for at least 5 min in a water bath (40 °C). Next, the stirring stopped to allow the mixture to separate into two layers. The upper aqueous phase was collected and precipitated with ethanol. The yellow precipitate was re-dissolved in water for further assembly of RP/CdS bio-nano-hybrid.

### Construction of RP/CdS bio-nano-hybrid and characterization

The RP strain was grown in 100 ml anaerobic UPM medium at 30 °C to an OD660 of 0.3. Then, the cells were collected by centrifugation and resuspended in fresh anaerobic UPM medium to the designed cell density. Next, 0.1 mM ligand-exchanged CdS QDs were added into the cell suspension and the mixture was cultivated at 30 °C with shaking (100 rpm) under anaerobic condition. After 12 h, the mixture was washed three times by anaerobic UPM medium in anaerobic workstation (Baker Ruskinn, Bridgend, UK), and the cell pellets were collected as RP/CdS bio-nano-hybrid. The viability of the RP/CdS bio-nano-hybrid cell was analyzed with fluorescence microscope (MF30, Guangzhou Mshot Photoelectric Technology CO., LTD., China) by using LIVE/DEAD BacLight bacterial viability kit (ThermoFisher, USA) staining. The RP/CdS bio-nano-hybrid cells were collected, pretreated and subjected for characterization with transmission electron microscope (TEM) (ht-7800, HITACHI, Japan), energy dispersive spectroscopy (EDS) (Team Octane Super, EDAX, USA), Raman (Thermo Fisher, USA), X-ray diffraction (XRD) (D8 Advance, Bruker, German) analyses.

### Photo-driven biomethanation

The RP and RP/CdS cells were collected by centrifugation and resuspended in the anaerobic NFM medium (Zhang et al. 2018) in an anaerobic workstation. For photocatalytic biomethanation (Ma et al. 2020a, b), NaHCO_3_ was added into the anaerobic NFM medium as the carbon source. L-Cystine (L-Cys) was added into the medium as sacrificial agent. Different wavelengths of light were obtained by using a xenon lamp with different optical filters. The biomethanation was conducted at 30 °C in anaerobic shaking incubator. Total protein of the cells was determined by using Bradford method (Ma et al. 2020a, b). The methane gas produced was detected by using a gas chromatograph (GC 7900, Techcomp, China) equipped with an 80/100 TDX-01 column (Ma et al. 2020a, b).

## Results and discussion

### Assembly of RP/CdS bio-nano-hybrid and characterization

**Fig.1:**
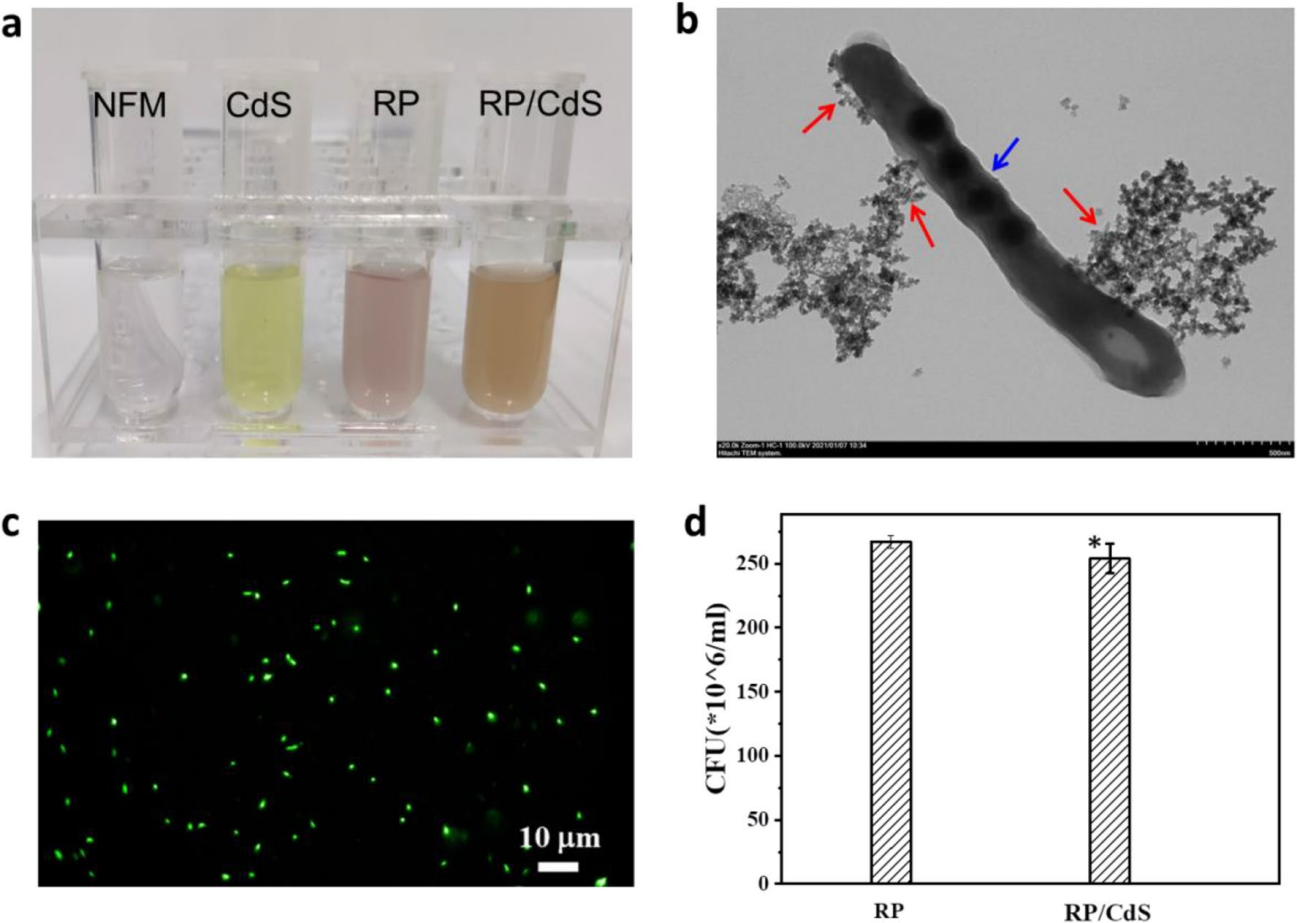
Assembly of RP/CdS bio-nano-hybrid cells. **a** The photographs of NFM medium, CdS QDs, RP cells suspension and RP/CdS hybrid cells suspension. **b** TEM image of the RP/CdS bio-nano-hybrid cells. Red arrows indicate the nanoparticles, blue arrow indicates the RP cell. **c** Fluorescence image of RP/CdS bio-nano-hybrid cell stained with LIVE-DEAD Baclight kit. Green fluorescence indicates living cell. **d** Colony formation unit of RP and RP/ CdS bio-nano-hybrid cells. Statistical significance of differences was analyzed by *t*-test, *p > 0.1 (RP/CdS *vs.* RP)

CdS is a commonly used nanoparticle for bio-nano-hybrid cells construction due to its suitable band gap for visible light response (Cheng et al. 2018). Thus, it is speculated to use CdS QDs to assemble the bio-nano-hybrid cells. First of all, the growth curve of the genetic engineered RP strain was characterized. It was found that this engineered RP strain showed typical growth curve with an exponential growth phase (0–96 h) and a steady growth phase (96–192 h) (Additional file 1: Fig. S1). The highest cell density of RP cell could reach an OD660 of ~ 0.35. The CdS QDs synthesized was also characterized by UV–vis and fluorescence analyses, which showed typical spectra of CdS nanoparticles (Additional file 1: Fig. S2). Next, the RP/CdS bio-nano-hybrid cells were constructed by incubating the RP cells with CdS QDs. The CdS QDs were anchored onto the cell surface due to electrostatic interaction. After that, the RP/CdS hybrid cells were isolated and the excessive CdS QDs in the suspension was washed (Fig. 1a). According to the TEM observation (Fig. 1b), it was clear that the RP/CdS hybrid cell consists of RP cell and nanoparticles. Many nanoparticles usually showed biotoxicity, which might be detrimental to microorganisms (Zhu et al. 2019). Thus, the cell viability of the RP/CdS hybrid was determined by using LIVE-DEAD Baclight staining and colony formation unit (CFU) analysis. As shown in Fig. 1c, the RP/CdS hybrid cells showed dominant green fluorescence, indicating high cell viability. According to the image analysis and statistical calculation, the cell viability of RP/CdS hybrid cells was about 98.9 ± 0.5%. Moreover, the CFU analysis indicated there was no statistically significant difference (*p* > 0.1) between bare RP cells and RP/CdS hybrid cells (Fig. 1d). Thus, these results suggested the RP/CdS bio-nano-hybrid cells with high cell viability were successfully constructed.

**Fig.2:**
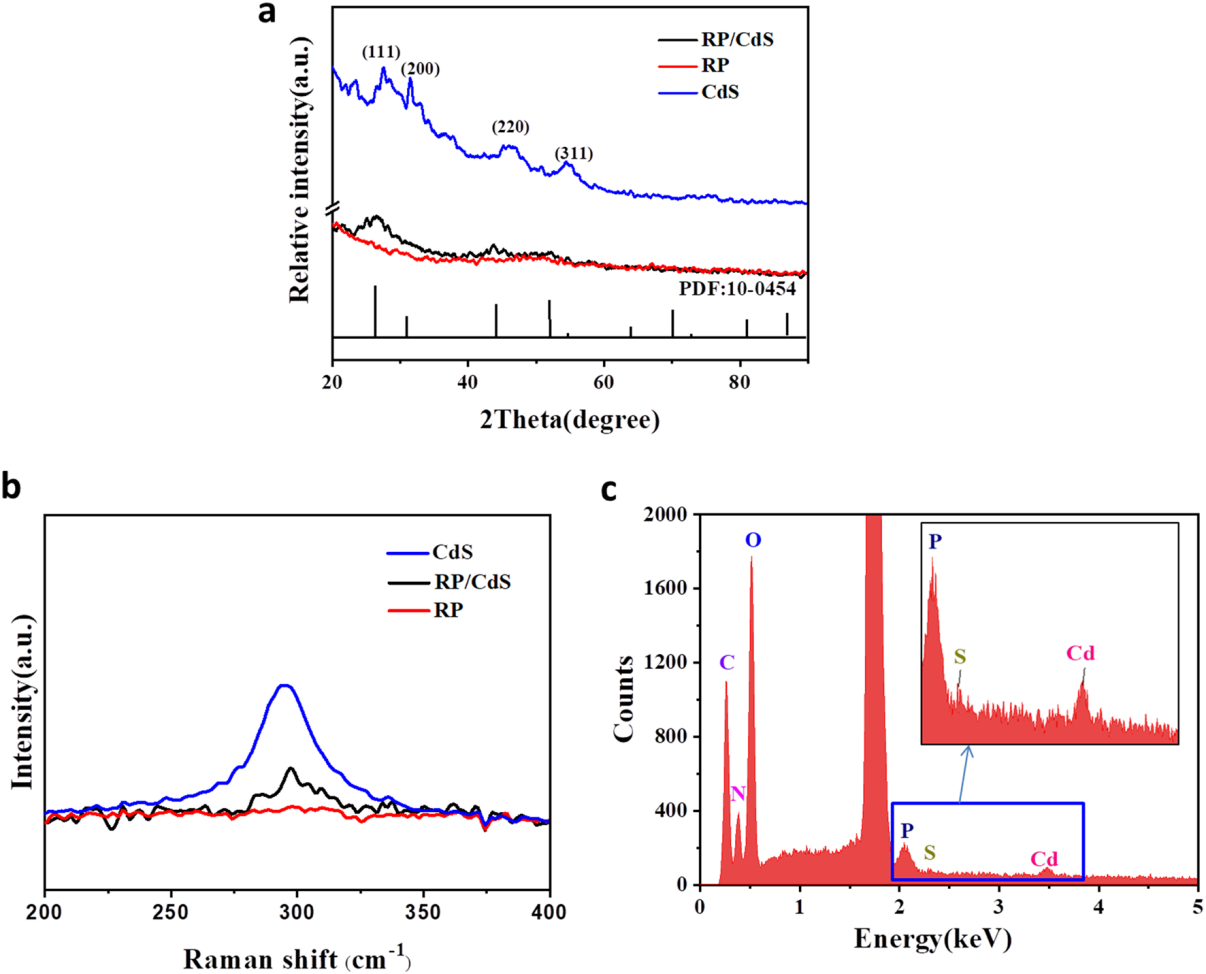
Physico-chemical characterization of RP/CdS bio-nano-hybrid cells. **a** XRD patterns of CdS QDs, RP cells and RP/CdS hybrid cells. The number in the image indicates the crystalline facet of the corresponding peak. **b** Raman spectra of CdS QDs, RP cells and RP/CdS hybrid cells. **c** EDS analysis of RP/CdS hybrid cells. The inset indicates the enlarged view of the selected area

To further confirm the formation of the RP/CdS bio-nano-hybrid cells, XRD, Raman and X-ray energy-dispersive spectroscopy (EDS) analyses were performed. It was observed that the pure CdS QDs showed typical diffraction peaks that could be indexed as (111), (220) and (311) planes of the cubic phase of CdS (JCPDS #10-0454) (Hao et al. 2019). The bare RP cells did not show any obvious XRD peaks. As expected, the XRD pattern of RP/CdS hybrid cells also showed diffraction peaks attributed to CdS QDs (Fig. 2a). In addition, Raman analysis of the RP/CdS hybrid cells also showed a typical peak similar to that of pure CdS, while bare RP cells did not exhibit obvious peak (Fig. 2b). The distinct Raman peak at ~ 295 cm^−1^ could be identified as the longitudinal optical phonon resulting from the Cd–S bond vibration (Ye et al. 2020; Ma et al. 2020a, b), suggesting the presence of CdS on the bio-nano-hybrid cells. Furthermore, EDS analysis was also applied to analysis the elemental composition of the RP/CdS hybrid cells. It could be concluded that Cd element and S element coexisted with C, N, O, P elements in these hybrid cells, although the Cd element and S element were not prominent due to their low content (the CdS loading in the hybrid cell is 0.11 wt%) (Fig. 2c). These results confirmed that the CdS QDs was assembled onto the RP cell, demonstrating that the RP/CdS bio-nano-hybrid cells were successfully constructed.

### Biomethanation performance of RP/CdS bio-nano-hybrid cells

**Fig.3:**
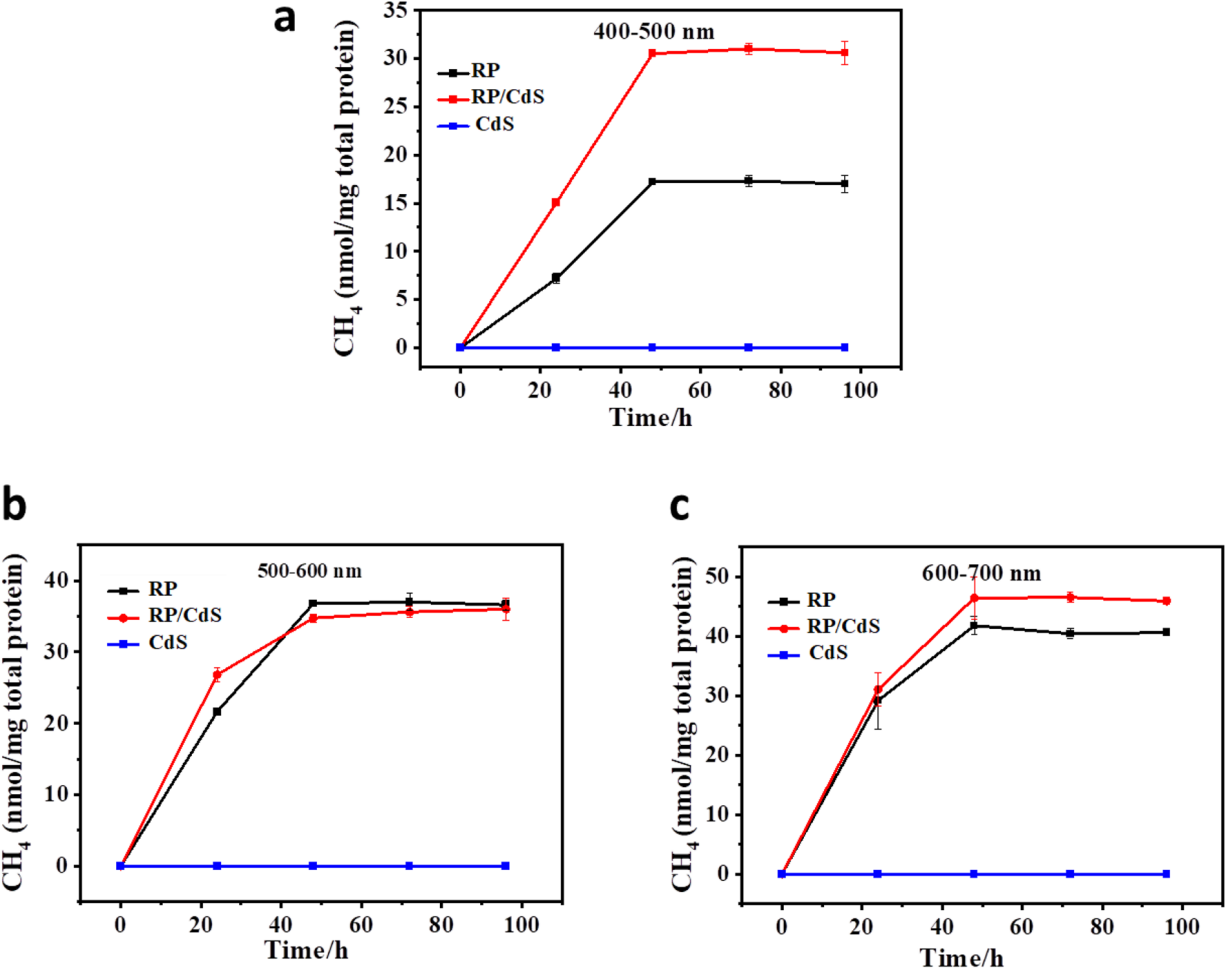
Biomethanation performance of RP cells and RP/CdS hybrid cells under the irradiation of light with different wavelength **a** 400–500 nm, **b** 500–600 nm, **c** 600–700 nm, respectively. The experiments were performed under OD660 = 0.5, 10 mM NaHCO_3_, 10 mM L-Cys and 100 lx light intensity

In order to improve the biomethanation efficiency of the RP cell under visible light, the methane production of the bio-nano-hybrid cells was compared with that of the bare RP cells. The visible light was divided into three different groups with different wavelength ranges by using different optical filters (400–500, 500–600, 600–700 nm). Once the RP cells or RP/CdS bio-nano-hybrid cells were anaerobically incubated under light irradiation, methane gas gradually accumulated and reached the highest production at around 48 h (Fig. 3). It was found that bare RP cells delivered the highest methane production of about 17, 36, and 42 nmol/mg total protein under irradiation with light of 400–500, 500–600, and 600–700 nm, respectively (Fig. 3). Pure CdS QDs under the same conditions did not produce detectable methane (Fig. 3). Then, the methane production by the RP/CdS bio-nano-hybrid cells was determined. As shown in Fig. 3a, under the irradiation of light RP/CdS bio-nano-hybrid cells with the wavelength of 400–500 nm, the RP/CdS bio-nano-hybrid cells reached the highest methane production of 31 ± 0.6 nmol/mg total protein, while the bare RP cells only showed the highest production of 17.3 ± 0.6 nmol/mg total protein. Under light irradiation with the wavelength of 500–600 nm, there was no significant difference could be observed between bare RP cells and the RP/CdS bio-nano-hybrid cells (Fig. 3b). Under the light irradiation with the wavelength of 600–700 nm, only a very slight improvement (< 10%) was observed by the RP/CdS bio-nano-hybrid cells as compared to the bare RP cells (Fig. 3c). In accordance, CdS QDs prepared here had high adsorption at 400–500 nm, while only weak adsorption was observed over 500 nm (Additional file 1: Fig. S2). Thus, it is reasonable that the modification of RP cells with CdS QDs greatly improved the biomethanation efficiency at 400–500 nm, while only slight effect was observed over 500 nm.

As only sodium bicarbonate was presence as the carbon source and no nitrogen source was provided in the biomethanation medium of the RP/CdS bio-nano-hybrid cells, the hybrid cells solely used CO_2_ for methane production (Fixen et al. 2018). In addition, it was already proved that the genetically engineered RP strain converted the CO_2_ to methane with a single enzymatic step catalyzed by the mutated nitrogenase (Fixen et al. 2018; Ma et al. 2020a, b). The biomethanation (CO_2_-to-CH_4_) by mutated nitrogenase of RP strain with the photogenerated ATP (energy source) and electrons (reducing equivalent) harvested from the reducing agents (Additional file 1: Fig. S3) (Fixen et al. 2018; Zheng et al. 2018). Here, it could be proposed that the CdS QDs generated photoelectrons under visible light irradiation, which might be involved in the biomethanation process via two possible pathways (Additional file 1: Fig. S3). On the one hand, the photogenerated electrons from CdS QDs might directly inject into the nitrogenase with cellular electron transfer pathways (Chen et al. 2019), which might directly reinforce the reducing equivalent for improved methane production. On the other hand, the photogenerated electrons from CdS QDs might be injected into cell, which in turn induce the proton efflux and facilitate ATP generation (Fixen et al. 2018). The enhanced ATP generation might further provide extra energy source to improve the efficiency of biomethanation. However, the detailed mechanism is still unclear, which calls for further investigation.

### Effects of different parameters on biomethanation of RP/CdS bio-nano-hybrid

With the aim of further improving the methane production of bio-nano-hybrid cells, the effects of different parameters (cell density, substrate concentration, L-Cys concentration, light intensity) on methane production of the RP/CdS bio-nano-hybrid cells were determined.

**Fig.4:**
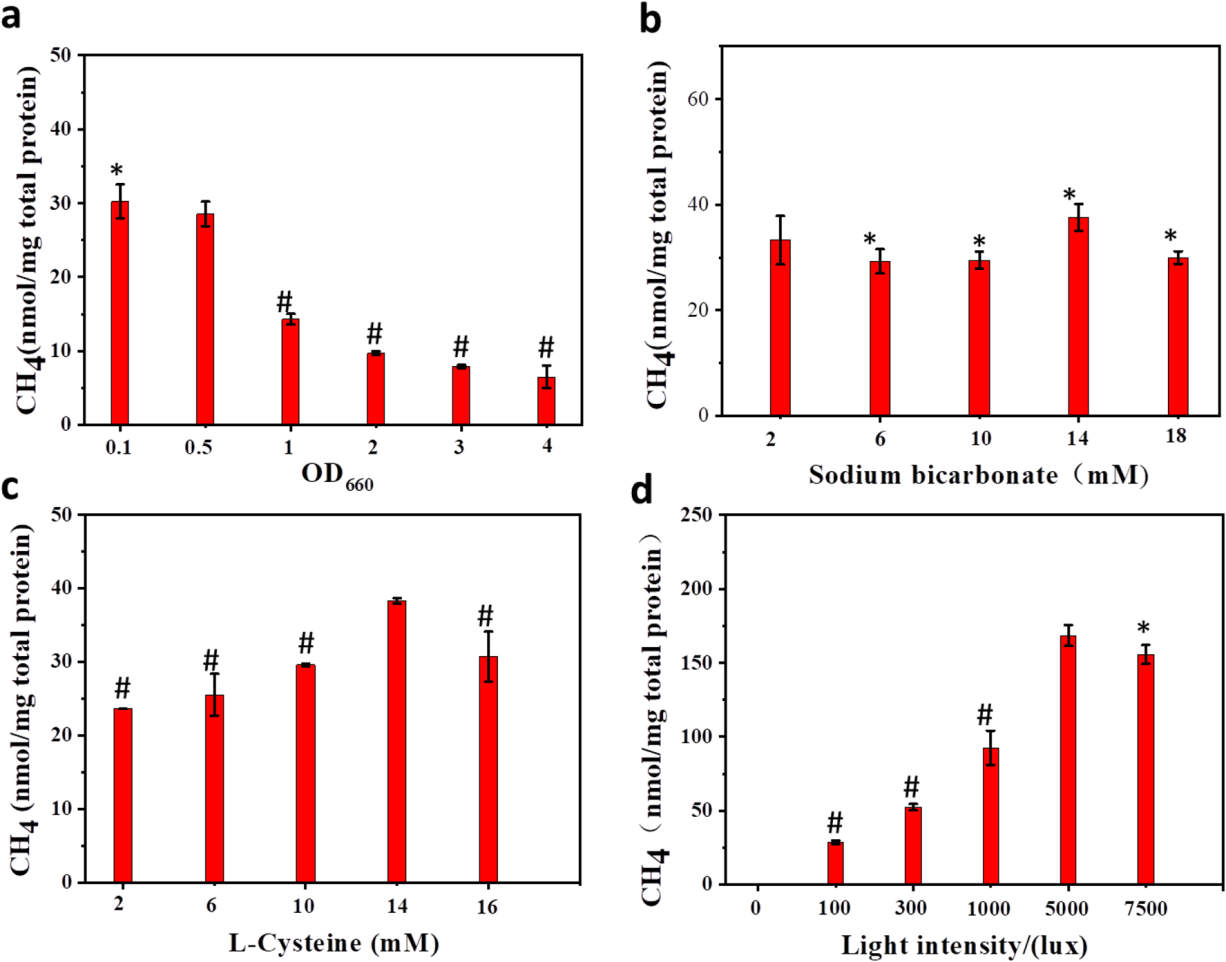
Effect of **a** cell density, **b** substrate concentration, **c** L-Cys concentration, and **d** light intensity on the biomethanation performance of RP/CdS hybrid cells. Apart from the tested parameter, other experimental conditions were the same with those used in Fig. 3. Statistical significance of differences was analyzed by *t*-test, *p > 0.1, #*p* < 0.01 (marked condition *vs.* unmarked condition)

For biological product synthesis by microorganisms, cell density and substrate concentrations are essential parameters. It was found that, cell density significantly affected the methane production from the RP/CdS hybrid cells (Fig. 4a). Once the initial cell density of OD660 was over 0.5, the methane production decreased along with the increased cell density. The methane production could reach 30 ± 2.3 nmol/mg total protein with the OD660 of 0.1, which is comparable with that obtained under the OD660 of 0.5 (*p* > 0.1). The effect of substrate (sodium bicarbonate) concentration on the methane production was also evaluated. According to the results (Fig. 4b), no significant difference (*p* > 0.1) could be found among these tested concentrations. The result was reasonable as the minimum concentration of substrate used here was already saturated for methane production by this RP/CdS hybrid cells.

**Fig. 5:**
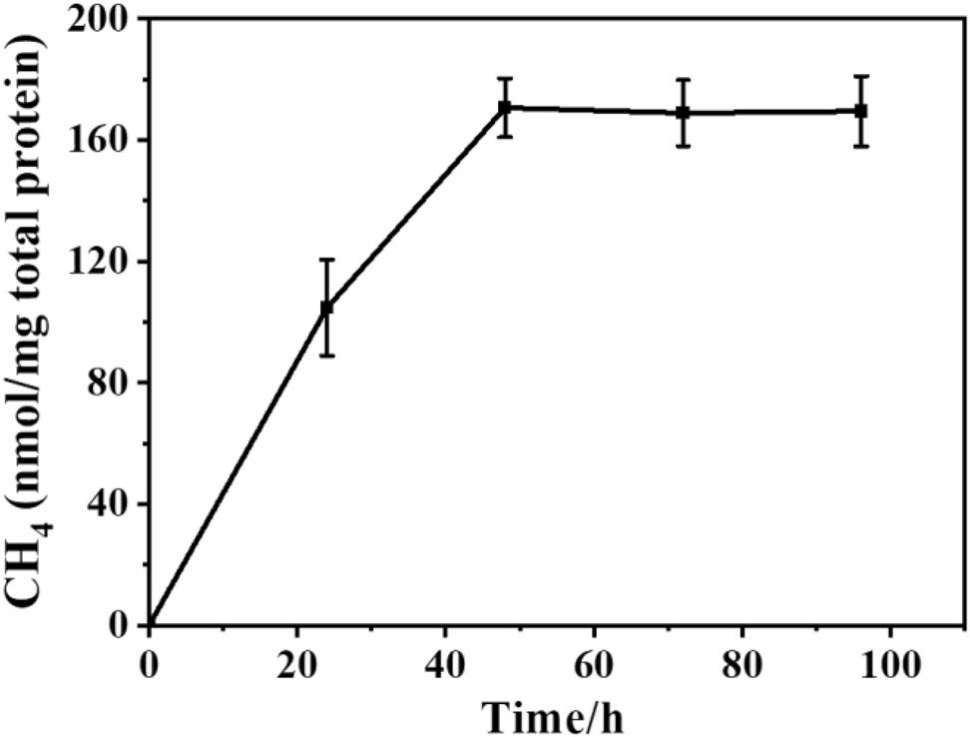
The biomethanation performance of RP/CdS hybrid cells under the optimized conditions (OD660 = 0.1, sodium bicarbonate = 2 mM, L-Cys = 14 mM, light intensity = 5000 lx)

It is also well-known that light intensity and scavenger concentration have great impact on the bio-photocatalysis (Jiang et al. 2018). The hole/radical scavenger could accelerate the separation of photogenerated charge and remove the detrimental radical to avoid the inactivation of bacterial cell (Shen et al. 2020). Therefore, the concentration of L-Cys should be optimized. With the increase of L-Cys concentration from 2 to 14 mM, the methane production increased from 24 nmol/mg total protein to 38 ± 0.4 nmol/mg total protein (Fig. 4c). The light intensity should also be optimized as low intensity cannot provide enough energy for high catalytic activity while high intensity may result in photo-toxicity (Jiang et al. 2018). As shown in Fig. 4d, the bio-nano-hybrid cells could not produce any methane without light irradiation. With the increasing of light intensity (100–5000 lx), the methane production dramatically increased along with the light intensity from about 30 nmol/mg total protein to 168 ± 7 nmol/mg total protein. However, further increasing the light intensity to 7500 lx resulted in decreased methane production, which might be due to photo-toxicity. Among these parameters, it could be found that the light intensity was the most prominent factor that dramatically affected the methane production. Based on the effects of different parameters, the methane production from RP/CdS hybrid cells was tested under the optimum conditions (OD660 = 0.1, sodium bicarbonate = 2 mM, L-Cys = 14 mM, light intensity = 5000 lx) (Fig. 5). It was found that the methane production steadily increased upon inoculation and reached the highest production of 171 ± 10 nmol/mg total protein.

## Conclusion

In summary, this work demonstrated the construction of bio-nano-hybrid bacterial cells for biomethane production from CO_2_ under visible light irradiation. The hybrid cells were assembled by using CdS QDs and the genetically engineered bacterial strain of *R. palustris*. The CdS QDs anchored on the cell surface might reinforce the visible light adsorption capability of *R. palustris* cell and thus facilitate the photo-driven biomethanation. Compared with the bare RP cells, the RP/CdS bio-nano-hybrid cells delivered ~ 79% higher methane production. Moreover, with essential parameters optimization, a methane production of 171 nmol/mg total protein was eventually reached under 400–500 nm visible light irradiation. This finding demonstrated the power of bio-nano-hybrid cells on photo-driven biomethanation, which would be helpful to extend the toolbox for methane production from CO_2_.

### Supplementary Information

**Additional file 1:** **Fig. S1. **The cell growth curve of RP strain in anaerobic UPM medium at 30 °C. **Fig. S2**. The **a** UV–vis spectrum and **b** fluorescence spectrum of the ligand-exchanged CdS QDs dispersed in water. The excitation wavelength in **b** is 400 nm. **Fig. S3**. Schematic of the photo-driven biomethanation by RP cell or RP/CdS hybrid cell.

## Data Availability

All data and materials are available in the main text and supporting information.

## Abbreviations

QDs: Quantum dots
RP: *Rhodopseudomonas palustris* CGMCC 1.2180 (*nifD*^V75AH201Q^)
L-Cys: L-Cystine
XRD: X-ray diffraction
EDS: Energy dispersive spectroscopy
TEM: Transmission electron microscope
CFU: Colony formation unit
