# Supplementary material for: Enhancement of photo-driven biomethanation under visible light by nano-engineering of Rhodopseudomonas palustris
Source: Bioresour Bioprocess. 2021 Apr 18;8(1):30. doi: 10.1186/s40643-021-00383-5 (PMC10992965; doi:10.1186/s40643-021-00383-5)
Supplement: Supplementary file 1 — Additional file 1: Fig. S1. The cell growth curve of RP strain in anaerobic UPM medium at 30 °C. Fig. S2. The a UV–vis spectrum and b fluorescence spectrum of the ligand-exchanged CdS QDs dispersed in water. The excitation wavelength in b is 400 nm. Fig. S3. Schematic of the photo-driven biomethanation by RP cell or RP/CdS hybrid cell. [file 40643_2021_383_MOESM1_ESM.docx]

# Supporting information

# Enhancement of photo-driven biomethanation under visible light by nano-engineering of *Rhodopseudomonas palustris*

Meng-Yuan Chen, Zhen Fang, Li-Xia Xu, Dao Zhou, Xue-Jin Yang, Hu-Jie Zhu, Yang-Chun Yong*

*Biofuels Institute, School of Environment and Safety Engineering, Jiangsu University, 301 Xuefu Road, Zhenjiang 212013, China*

*Corresponding author, Email: ycyong@ujs.edu.cn

**Culture medium**

The compositions of the media used in this study are listed in the following,

YP medium: 3 g/L yeast extract, 3 g/L tryptone, pH 6.8

NFM medium: 0.58 g/L MgSO_4_, 0.067 g/L CaCl_2_, 2 mg/L FeSO_4_, 6.8 g/L KH_2_PO_4_, 18 g/L Na_2_HPO_4_▪_12_H_2_O, 1 mL trace elements solution

UPM medium: 0.58 g/L MgSO_4_, 0.067 g/L CaCl_2_, 2 mg/L FeSO_4_, 6.8 g/L KH_2_PO_4_, 18 g/L Na_2_HPO_4_▪_12_H_2_O, 1 mL trace elements solution, 1 g/L yeast extract and 3 g/L sodium acetate

Trace elements solution: 0.25 g EDTA, 1.095 g ZnSO4▪7H2O, 0.5 g FeSO4▪7H2O, 154 mg MnSO4▪H2O, 39.2 mg CuSO4▪5H2O, 24.8 mg Co(NO2)2▪6H2O, and 17.7 mg Na2B2O7▪10H2O


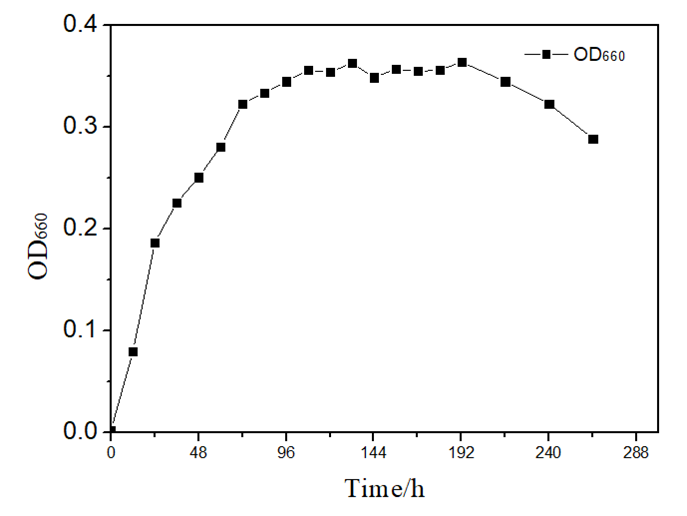


**Fig. S1.** The cell growth curve of RP strain in anaerobic UPM medium at 30°C.


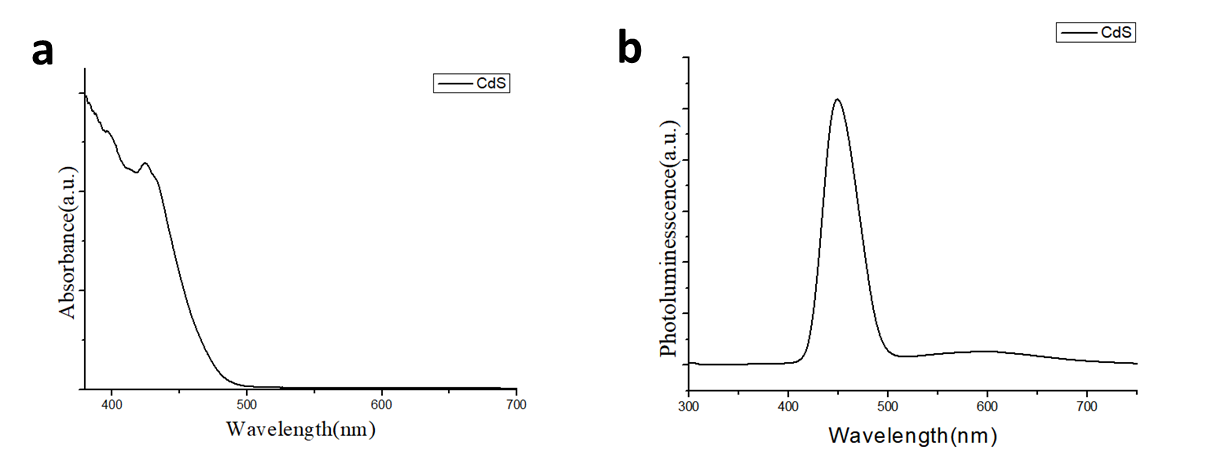


**Fig. S2**. The (a) UV-vis spectrum and (b) fluorescence spectrum of the ligand exchanged CdS QDs dispersed in water. The excitation wavelength in (b) is 400 nm.


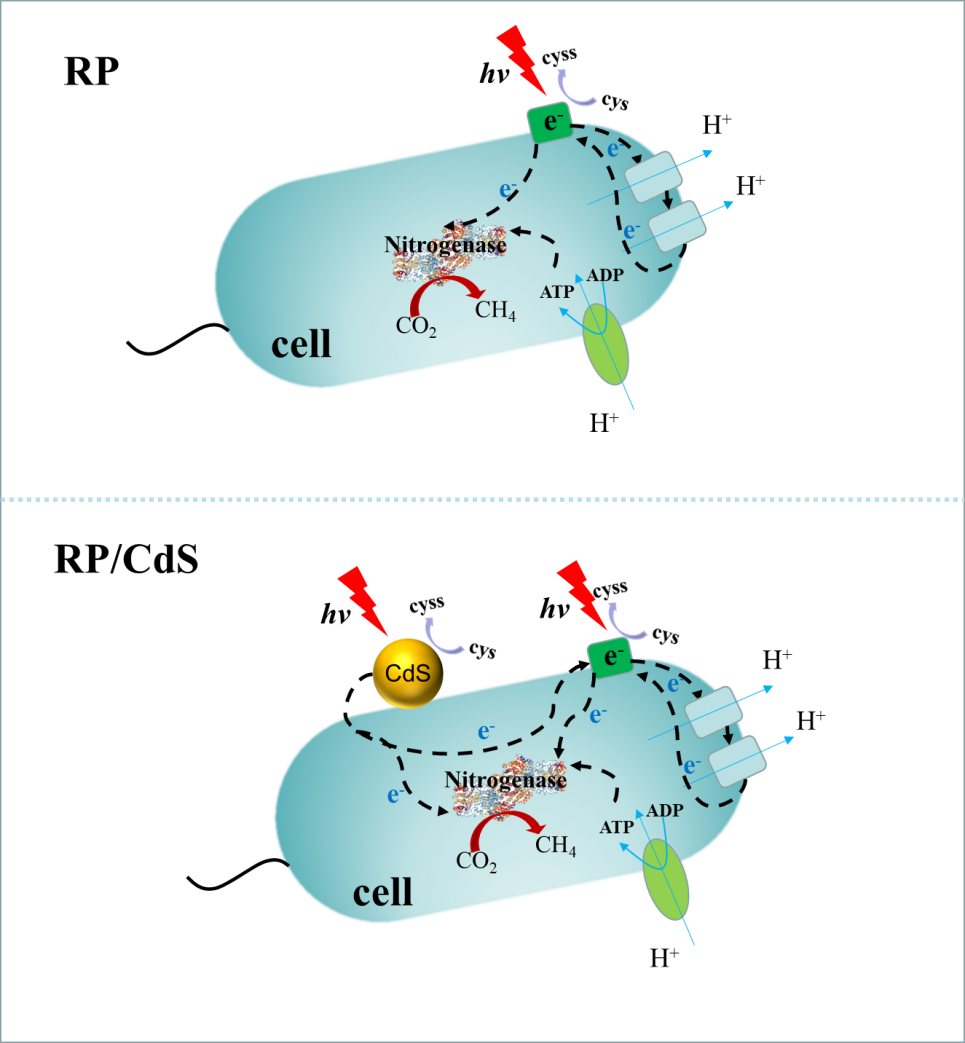


**Fig. S3**. Schematic of the photo-driven biomethanation by RP cell or RP/CdS hybrid cell.
